# Neurogenic and Neurotrophic Effects of BDNF Peptides in Mouse Hippocampal Primary Neuronal Cell Cultures

**DOI:** 10.1371/journal.pone.0053596

**Published:** 2013-01-08

**Authors:** Maria del Carmen Cardenas-Aguayo, Syed Faraz Kazim, Inge Grundke-Iqbal, Khalid Iqbal

**Affiliations:** 1 Department of Neurochemistry, New York State Institute for Basic Research in Developmental Disabilities, Staten Island, New York, United States of America; 2 Neural and Behavioral Science Graduate Program, State University of New York Downstate Medical Center, Brooklyn, New York, United States of America; Nathan Kline Institute and New York University School of Medicine, United States of America

## Abstract

The level of brain-derived neurotrophic factor (BDNF), a member of the neurotrophin family, is down regulated in Alzheimer’s disease (AD), Parkinson’s disease (PD), depression, stress, and anxiety; conversely the level of this neurotrophin is increased in autism spectrum disorders. Thus, modulating the level of BDNF can be a potential therapeutic approach for nervous system pathologies. In the present study, we designed five different tetra peptides (peptides B-1 to B-5) corresponding to different active regions of BDNF. These tetra peptides were found to be non-toxic, and they induced the expression of neuronal markers in mouse embryonic day 18 (E18) primary hippocampal neuronal cultures. Additionally, peptide B-5 induced the expression of BDNF and its receptor, TrkB, suggesting a positive feedback mechanism. The BDNF peptides induced only a moderate activation (phosphorylation at Tyr 706) of the TrkB receptor, which could be blocked by the Trk’s inhibitor, K252a. Peptide B-3, when combined with BDNF, potentiated the survival effect of this neurotrophin on H_2_O_2_-treated E18 hippocampal cells. Peptides B-3 and B-5 were found to work as partial agonists and as partial antagonists competing with BDNF to activate the TrkB receptor in a dose-dependent manner. Taken together, these results suggest that the described BDNF tetra peptides are neurotrophic, can modulate BDNF signaling in a partial agonist/antagonist way, and offer a novel therapeutic approach to neural pathologies where BDNF levels are dysregulated.

## Introduction

Brain derived neurotrophic factor (BDNF), a member of the neurotrophin family that also includes nerve growth factor (NGF), neurotrophin-3 (NT-3) and neurotrophin-4/5 (NT-4/5), promotes neuronal survival, differentiation, and synaptic function [1], [2] through the signaling of its receptor tropomyosin-related kinase-B (TrkB). Brain derived neurotrophic factor is of particular therapeutic interest because its expression level is altered in many neurological disorders. A neurotrophic factor starvation, including NGF and BDNF deficiency, that begins in the early stages of Alzheimer disease (AD) and ultimately causes neuronal degeneration, cell death, and loss of cholinergic neurotransmission in the late stages of the disease has been reported [3]–[7]. Additionally, the expression level of BDNF is also reported to be reduced in Parkinson’s disease (PD), depression, and stress [8], [9]. Conversely, autism spectrum disorders (ASDs) are characterized by an increase in BDNF level [10]. Thus, modulation of BDNF level in these neurological disorders as a potential therapeutic approach is suggested. A number of properties limit the therapeutic use of BDNF, such as it’s very short (less than 1 min) plasma half-life, and it’s poor blood brain barrier (BBB) and intraparenchymal penetrations [11]. Thus, the use of molecules such as small peptides that could mimic or modulate the functions of BDNF, and have higher permeability and stability than BDNF itself, serve as an attractive alternative approach.

Brain derived neurotrophic factor plays important roles in the plasticity of several regions of the central nervous system (CNS) during development, adulthood, and ageing. The multiple roles of BDNF depend on functional and morphological changes, like protein phosphorylation, generation of new neurons, and cytoskeletal reorganization of dendritic spines. In hippocampal neurons, cyclic adenosine monophosphate (cAMP) controls BDNF-induced TrkB phosphorylation and dendritic spine formation by modulating the signaling and trafficking of TrkB [5], [12].

Brain derived neurotrophic factor shares about 50% amino acid identities with NGF, NT-3 and NT-4/5. Each neurotrophin consists of a non-covalently-linked homodimer, and contains a signal peptide following the initiation codon and a proregion containing an N-linked glycosylation site [13]. Initially neurotrophins are produced as proneurotrophins (molecular weight∼30 KDa), that are cleaved by enzymes such as prohormone convertases e.g. furin generating the mature neurotrophin (molecular weight∼14–26 KDa) [13], [14]. Proneurotrophins have distinct biological activities and binding characteristics [13].

The immature form of BDNF is called proBDNF, and it consists of 247 amino acids (in comparison with the mature form of BDNF that has 119 amino acids). This proneurotrophin binds a different receptor, known as low affinity p75NGFR, a member of the tumor necrosis factor (TNF) receptor super family [1], [15], and minimally binds Trk receptors. Brain derived neurotrophic factor and proBDNF are reported to have opposite effects. The activation of p75NGFR receptor can cause apoptosis in a variety of systems [16]; instead, the activation of the TrkB receptor alone, as mentioned above, can promote differentiation, survival, and/or neuronal plasticity. In physiological conditions, neurons probably do not have high amounts of available extracellular proBDNF because the endogenous proBDNF is rapidly converted to BDNF [17]. Nevertheless, this concept is currently controversial as a recent study by Yang et al argues that proBDNF is not a transient biosynthetic intermediate and mouse neurons secrete both proBDNF and mature BDNF [18]. They observed highest levels of proBDNF perinatally which declined but were still detectable in adulthood.

Pharmacologic modulation of BDNF levels has been suggested as a potential treatment strategy for human neurodegenerative diseases [19]. The general lack of success of neurotrophic factors in clinical trials (due to low stability in plasma and low permeability through the BBB) has led to the idea that low molecular weight neurotrophic factor mimetics can serve better as pharmacological agents [20], [21].

The aim of the current study was to identify, through the use of neutralizing antibodies directed to the active sites of BDNF, a group of peptides harboring part of the sequence of BDNF (particularly of the active site), and to evaluate the capability of these peptides to mimic the function of BDNF. The possible neurogenic/neurotrophic potential of these peptides was evaluated in mouse hippocampal primary neuronal cell cultures. The comparative effects of these peptides were also studied to find the most active ones that could be developed as therapeutic drugs, as such or after appropriate modifications, in animal models of neurological diseases in the future. In this study, we report the results of the initial screening of the synthesized BDNF peptides and the evaluation of whether they could modulate the BDNF function in an in-vitro model.

## Materials and Methods

### Design and Selection of BDNF Peptides

#### Employing epitope mapping of neutralizing antibodies to human BDNF

**Figure 1:**
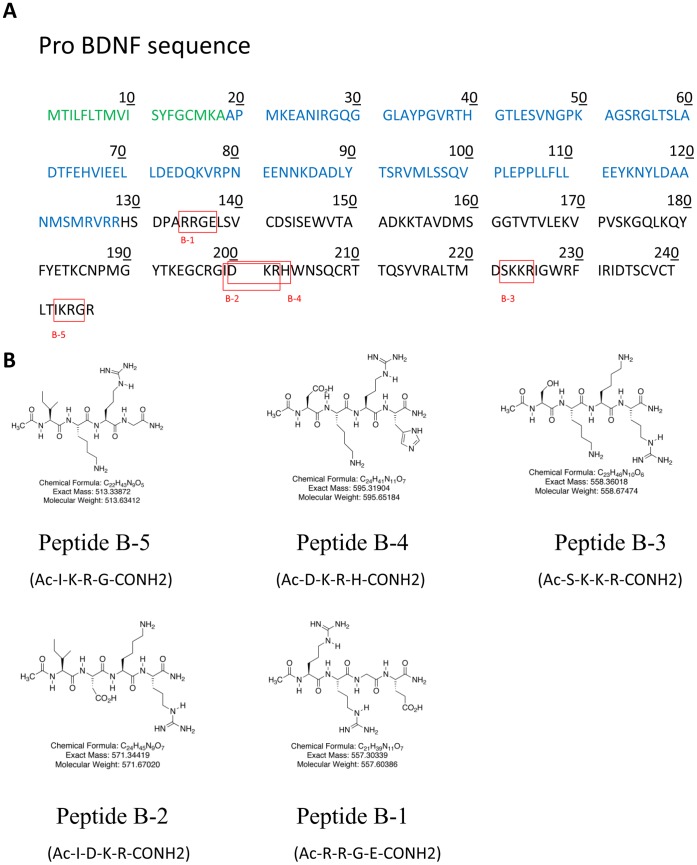
BDNF tetrapeptides used in this study. (A) Amino acid sequence of human BDNF. The signal peptide (18 amino acids, positions 1–18) is shown in green, the propeptide (110 amino acids, positions 19–128) in blue, and the BDNF sequence, (119 amino acids, positions 129–247, molecular weight 26 kDa) in black. The sequences of the 5 tetra peptides used in this study (B-1 to B-5) are boxed (as red squares). (B) Chemical structures of the BDNF peptides used in this study.

(http://uniprot.org/uniprot/P23560), we identified different active regions of BDNF. We then narrowed down the active regions, and synthesized five druggable tetra peptides corresponding to amino acid residues 6–9, 71–74, 72–75, 94–97 and 115–118 of human BDNF (Figure 1A and B). Peptides B-1 to B-5 were N-terminally acetylated and C-terminally amidated (Fig. 1B). The sequences of these peptides are as follows: Peptide B-5 (Ac-I-K-R-G-CONH2 corresponding to amino acids (AAs) 243–246 of pro BDNF and AAs 115–118 of BDNF), molecular weight 513.63; Peptide B- 4 (Ac-D-K-R-H-CONH2 corresponding to AAs 200–203 of pro BDNF and AAs 72–75 of BDNF), molecular weight 595.65; Peptide B-3 (Ac-S-K-K-R-CONH2 corresponding to AAs 222–225 of pro BDNF and AAs 94–97 of BDNF), molecular weight 558.67; Peptide B-2 (Ac-I-D-K-R-CONH2 corresponding to AAs 193–196 of pro BDNF and AAs 71–74 of BDNF), molecular weight 571.67; and Peptide B-1(Ac-R-R-G-E-CONH2 corresponding to AAs 134–137 of pro BDNF and AAs 6–9 of BDNF), molecular weight 557.6 (Fig. 1A). These peptides were synthesized at Stanford University Protein and Nucleic Acid Research Lab on a commercial basis. All peptides were purified by HPLC to 95–99% of purity and their identity was confirmed by mass spectrometry. The synthetic peptides were soluble in water.

### Primary Cell Culture

To screen these peptides, we used primary neuronal cell cultures from embryonic day 18 (E18) C57BL/6 mouse hippocampus. At 4th day in vitro (DIV), the culture medium was exchanged with the medium containing the peptides. The primary neuronal cultures were established as previously reported [22]. Briefly, C57BL/6 time pregnant E18 female mice from Charles River Labs were anesthetized and killed by cervical dislocation. All studies were performed in accordance with the recommendations in the Guide for the Care and Use of Laboratory Animals of the National Institutes of Health (NIH). The protocol was approved by the Institutional Animal Care and Use Committee (IACUC) of the NYS Institute for Basic Research in Developmental Disabilities (Protocol no. 199). All surgeries were performed under anesthesia, and all efforts were made to minimize suffering. Embryos were removed and placed in cold Hibernate A (Brain bits, Springfield, IL, USA), and all of the following steps were performed in ice-cold Hibernate A, under a stereoscopic (dissection) microscope placed in a laminar flow hood. Fetal brains were removed carefully and fore brain were separated. Then the hippocampus, including the cortex surrounding the area of the hippocampus, was dissected and cut into small pieces using microsurgical scissors. The cut tissue was transferred with number 5 forceps to 15 ml tubes containing 0.1% trypsin in Versene (Invitrogen Life Technologies, Grand Island, NY, USA) and incubated for 15 min at 37°C followed by inactivation with 10% fetal bovine serum (FBS) in Neurobasal complete medium (Neurobasal Medium supplemented with 2×B-27, 0.30% glutamine, and penicillin/streptomycin 0.1 mg/ml and 0.1 U/ml respectively) All medium components were purchased from Invitrogen, Grand Island, NY, USA. Every 72 hours the medium was replaced and supplemented with fresh medium with or without a test peptide. Cells were maintained in an incubator at 37°C at 5% CO_2_/95% atmospheric air.

For recovering proteins, cells were seeded in 6-well dishes coated with poly-D-lysine (Sigma-Aldrich, St. Louis, MO, USA), 50 µg/ml for an overnight, at a density of 1×10^6^ cells/well. For immunocytochemistry, cells were seeded onto 5 mm cover slips coated with poly-D-lysine at a density of 7×10^4^ cells/well in 100 µl Defined Medium in 96 well plates.

The cells were cultured for four days in vitro prior to the beginning of the treatment with the peptides. The treatment with the peptides was done for 5 days (starting at DIV 4 and finishing at DIV 9). The culture medium was exchanged completely at the beginning of the treatment with a test peptide (at DIV4), and every 72 h until DIV 9.

### Cell Lines

To study the activation of the TrkB receptor with BDNF and/or with the peptides, we used NIH 3T3 cells (obtained from ATCC, Rockville, MD) stably transfected with the TrkB receptor (a kind gift from Dr. David Kaplan; Montréal Neurological Institute, Montréal, Québec, Canada [23]. The cell culture medium used for this cell line was: DMEM-high glucose, 10% normal calf serum, 1% glutamax, 1% sodium pyruvate, 1% penicillin-streptomycin; supplemented with the selection antibiotic G418 in a final concentration of 100 µg/ml (to guarantee the stable expression of the Trkb transgene). All Medium components were purchase from Gibco Life Technologies-Invitrogen, Grand Island, NY, USA. For all the experiments using the cell line NIH3T3-TrkB, the medium containing serum was used only for expansion of the cell line. At the beginning of each experiment cells were washed twice with medium without serum, and the experiments were performed in the absence of serum to avoid endogenous BDNF levels present in the serum (17, 18). Fibroblast cells, themselves, do not express or secrete BDNF.

### Peptide Treatment

Peptides were dissolved in water in serial dilutions of 10 mM, 1 mM, 100 µM and 10 µM from which the necessary amount was added directly to the culture medium to a final concentration of 0.05 µM, 1 µM, or 10 µM. The BDNF (Peprotech, NJ, USA) was used as a reference in concentrations of 20 ng/ml (0.79 nM) and 100 ng/ml (3.95 nM).

### Use of Inhibitors

Cycloheximide (CHX) (Sigma-Aldrich, St Louis, MO, USA) was used for blocking protein synthesis. CHX original stock was 100 mg/ml (355.4 mM) in DMSO; from a second stock of 35.54 mM in DMSO, the dilutions were made to the final concentrations of 100 µM. The K252a (CALBIOCHEM/EMD) was used to inhibit the TrkB Receptor. From a stock of 100 µM in DMSO, K252a was diluted to a final working concentration of 200 nM. The E18 primary hippocampal cells were pre-treated with CHX or K252a 1 h prior to the addition of the peptides or BDNF and a time course of these treatments for 15 min, 60 min and 2 days was studied.

### Immunocytochemistry

After 5 days of treatment, cells seeded on cover slips were fixed in 4% paraformaldehyde (Electron Microscopy Sciences, PA, USA) for 30 min at room temperature, and then washed 2 times in PBS for storage at 4°C prior to staining. Cells were permeabilized in 0.2% Triton-X-100 in PBS for 30 min at 25°C and incubated in blocking buffer (1% BSA w/v, 0.2% Triton- X-100 v/v in PBS) for 60 min at 25°C. The cells were then incubated with primary antibodies at the appropriate dilutions in blocking buffer at 4°C for 16 h, washed 3 times for 10 min with 0.2% Triton X-100 in PBS, and incubated with fluorescently-labeled secondary antibodies diluted in blocking buffer for 1 h at 25°C in the dark. Cover slips were washed 3×10 min in 0.2% Triton X-100 in PBS and mounted on glass slides with GelMount (Biomeda, Foster City, CA, USA). The following primary antibodies were used: rabbit polyclonal anti-GFAP (astrocytic marker, 1∶500; Sigma-Aldrich, St. Louis, MO, USA); rabbit polyclonal anti-neurofilament-M (NFM) (early neuronal marker, 1∶200; Chemicon/Millipore, Billerica, MA, USA); mouse monoclonal anti-NeuN (late neuronal marker, 1∶300; Millipore, Billerica, MA, USA); rabbit polyclonal anti-Tuj-1, β–III- tubulin (early neuronal marker, 1∶800; Covance, Emeryville, CA, USA); mouse monoclonal SMI 52 to MAP-2 (dendritic marker, 1∶1000; Covance, Emeryville, CA, USA); rabbit polyclonal anti-synapsin I (pre-synaptic marker, 1∶2000; Stressgen, Farmingdale, NY, USA); and rabbit monoclonal anti-PSD95 (post-synaptic marker, 1∶100; Cell Signaling, Danvers, MA, USA). Secondary antibodies goat anti-mouse and anti-rabbit IgGs conjugated with AlexaFluor 594 were used at 1∶1000 (Molecular Probes, Carlsbad, CA, USA). The nuclei were stained with 1 µM TOPRO in PBS (Invitrogen, Grand Island, NY, USA). Mounted cover slips were examined using 40x oil immersion objective of a Nikon 90i fluorescent microscope equipped with Nikon C1 three-laser confocal system and a Nikon DS U1 digital camera, and analyzed with EZ-C1 Viewer Image software, Version 6.0. The software IMAGE J (NIH, version 1.46 r) was used to measure the intensity of fluorescence (using the commands Analyze and then Measure, to obtain the Integrated Density values) of the confocal images. Additionally, rainbow scale images were obtained from the confocal images using the software IMAGE J (NIH, version 1.46 r) to confirm the increase in the level of expression of the neuronal markers analyzed in the study. In the rainbow images, the warm colors (like red and yellow) represent the higher level of expression of the neuronal marker analyzed, and the cold colors (like blue) represent the lowest level of expression of the marker. White represents the highest level of expression whereas black represents no expression at all.

### Western Blots

Following treatment in 6-well plates, cells were washed 2 times in glucose buffered saline, GBS (5.4 mM KCl, 138 mM NaCl, 22 mM glucose, and 2 mM Na-KPO pH 7.2), and then lysed by 5 min incubation on ice in 100 or 150 µl of ice-cold RIPA buffer (PBS, 1% w/v NP-40 from Fisher Scientific, 0.1% w/v SDS, and 0.5% w/v sodium deoxycholate) containing 1 mM AEBSF (Gold Biotechnology, St. Louis, MO, USA), 10 µg/ml aprotinin (Sigma-Aldrich, St. Louis, MO, USA), and 20 µg/ml of leupeptin and pepstatin (US Biochemicals, Cleveland, OH, USA), and phosphatase inhibitors: NaF, Na orthovanadate, β-glycerophosphate, and microcystein (Sigma-Aldrich, St. Louis, MO, USA). Extracts were prepared by collecting and pooling a minimum of 2 wells by scraping, and lysates were centrifuged at 20,000×g for 10 min at 4°C. Protein concentration of each cell lysate was determined using the BCA kit (Thermo Scientific, Rockford, IL, USA). The lysates (7.5–20 µg total protein) were separated on 10% SDS-PAGE gels (except for BDNF where 12% gels were employed) and transferred to 0.45 µm PVDF membrane (Pall, Pensacola, FL, USA) for probing with antibodies as noted. Blots were blocked for 1 hr at 37°C in TBST (0.05% Tween 20 in TBS) containing 5% w/v blotting grade dry milk (Bio-rad, Hercules, CA, USA), incubated in primary antibody overnight in blocking buffer at 4°C, washed 3 times for 10 min in TBST at room temperature, followed by incubation with secondary antibody i.e. Peroxidase-conjugated anti-mouse or anti rabbit IgG (Jackson ImmunoResearch Laboratories, West Grove, PA, USA) diluted in blocking buffer. Blots were washed 3×10 min in TBST and immunoreactive protein bands were visualized with enhanced chemiluminescence (ECL) reagents (Pierce, Rockford, IL, USA). The ECL films of the blots were scanned and analyzed using Multi Gauge software version 3.0 (Fujifilm, Tokyo, Japan).

The following primary antibodies were used: rabbit polyclonal anti-NFM (early neuronal marker, 1∶1000; Chemicon/Millipore, Billerica, MA, USA); mouse monoclonal anti-NeuN, clone A60 (late neuronal marker, 1∶500; Millipore, Billerica, MA, USA); rabbit polyclonal anti-Tuj-1, β–III-tubulin (early neuronal marker, Covance; 1∶800, Emeryville, CA, USA); mouse monoclonal SMI 52 to MAP-2 (dendritic marker, 1∶1000; Covance, Emeryvilly, CA, USA); rabbit polyclonal anti-Synapsin I (pre-synaptic marker, 1∶2000; Stressgen, Farmingdale, NY, USA); rabbit monoclonal anti- PSD95 (post-synaptic marker, 1∶1000; Cell signaling, Danvers, MA, USA); anti tau 92e (axonal marker, 1∶10000; [24], [25]; rabbit polyclonal anti- BDNF (N-20) (1∶200 Santa Cruz Biotechnology, Santa Cruz, CA, USA ); rabbit polyclonal anti-TrkB (total) (1∶500; Santa Cruz Biotechnology, Santa Cruz, CA, USA); rabbit polyclonal anti- pTrkB (Tyr706) (1∶400; Santa Cruz Biotechnology, Santa Cruz, CA, USA), TrkC (1∶1000; Cruz Biotechnology, Santa Cruz, CA, USA); and pTrkY490 (1∶500; Upstate USA Inc., Charlottesville, VA, USA). For loading control, the blots were developed with rabbit polyclonal antibody to GAPDH (1∶2000; Santa Cruz Biotechnology, Santa Cruz, CA, USA).

### Cell Death and Cell Viability

Evaluation of cell death and cell viability was performed using the LDH cytotoxicity assay kit (Promega, Madison WI, USA), following manufacturer’s instructions. Percentages of cell death and cell viability were plotted separately.

### Statistical Analysis

The statistical analyses were conducted using SPSS version 16.0 (© SPSS Inc., 1989–2007, Chicago, Illinois, USA), Sigma Plot version 7.0 (San Jose, CA, USA), and GraphPad Prism version 5.0 (GraphPad software inc., La Jolla, CA, USA). Data are presented as mean±standard deviation. For analysis involving multiple groups, one-way ANOVA with *post* hoc Fischer’s or Tukey’s, or Bonferroni’s test (as appropriate) was used. For all other comparisons (including inter-group comparisons), Student’s *t*-test was used. For all purposes, *p*<0.05 was considered as statistically significant.

## Results

### BDNF Peptides are Non-toxic to Neural Cells in vitro

**Figure 2:**
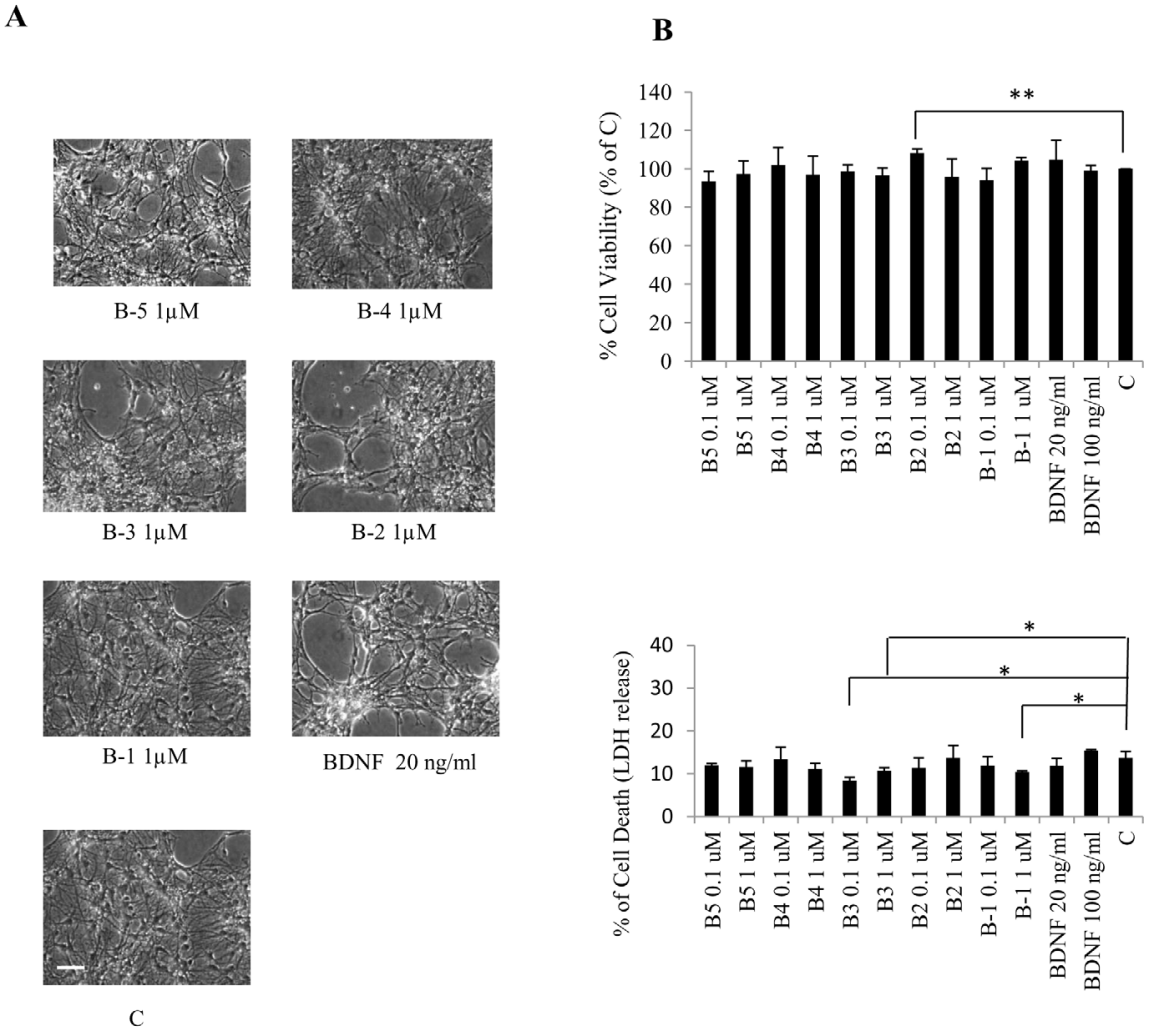
BDNF peptides B-1 to B-5 are non-toxic to E18 mouse primary hippocampal neuronal cells. (A) Phase contrast photomicrographs of hippocampal neurons vehicle-treated or treated with BDNF (20 ng/ml) or the indicated peptides (at 1 µM) for 5 days. Scale bar represents 20 µm. (B) Effect of BDNF peptides on neuronal survival. Lactate dehydrogenase (LDH) assay showing the percentages of cell viability and cell death after 5 days of treatment with the peptides (at 0.1 or 1 µM) or BDNF (at 20 and 100 ng/ml; 0.79 and 3.95 nM respectively). Counts were normalized to survival achieved with vehicle treatment for 5 days (control, C). Values for each concentration derived from 3 independent experiments. *p<0.05, **p<0.01. ANOVA with post-hoc test and/or Student’s *t* test.

To study any toxic effect of the peptides, mouse E18 primary hippocampal neurons were treated with the five BDNF peptides individually for up to 5 days at different doses [5 nM, 25 nM, 50 nM (data not shown), 100 nM (0.1 µM), 1000 nM (1 µM), and 10,000 nM (10 µM)]. Phase contrast photomicrographs revealed no gross morphological changes in cells treated with the BDNF peptides, as compared to the vehicle-treated or BDNF-treated cells (Fig. 2A). Accordingly, there was no significant change in the viability of cells treated with any of the peptides as evaluated by the lactate dehydrogenase (LDH) assay (Fig. 2B). The only exception was peptide B-2 0.1 µM which significantly increased cell viability compared to vehicle treated cells (control, C) (Student’s *t* test, *p* = 0.0092). Also, there was a significant reduction in cell death when cells were treated with Peptide B-3 (0.1 µM, ANOVA, *p* = 0.012, post-hoc test, *p*<0.001, Student’s *t* test, *p* = 0.0061; 1 µM, ANOVA, *p* = 0.012, post-hoc test, *p* = 0.033, Student’s *t* test, *p* = 0.0398) and Peptide B-1 (1 µM, ANOVA, *p* = 0.012, post-hoc test, *p* = 0.01, Student’s *t* test, *p* = 0.0239) in comparison to vehicle treated cells (control, C). Thus, the five BDNF peptides were not toxic for the E18 hippocampal cells. Initial biochemical evaluation of the effect of BDNF peptides did not show any significant effect of peptides B-2 and B-1 on the expression of neuronal markers, β-III-tubulin and NeuN, and synaptic marker, synapsin I in hippocampal neural precursor cells (NPCs) from E18 mice (Fig. S1). Thus, we selected only 3 peptides (B-5, B-4 and B-3) for further characterization.

It is appropriate to mention here the fact that in all the experiments using primary hippocampal neurons, the presence of endogenous BDNF is expected in all conditions assayed, since hippocampal cells are known to secrete this growth factor (17, 18). However, experiments using NIH3T3 cells were performed in the absence of serum to avoid the confounding effect of endogenous BDNF and other growth factors present in the serum. Fibroblasts cells, themselves, normally do not express BDNF.

### BDNF Peptides Induce the Expression of Neuronal Markers

**Figure 3:**
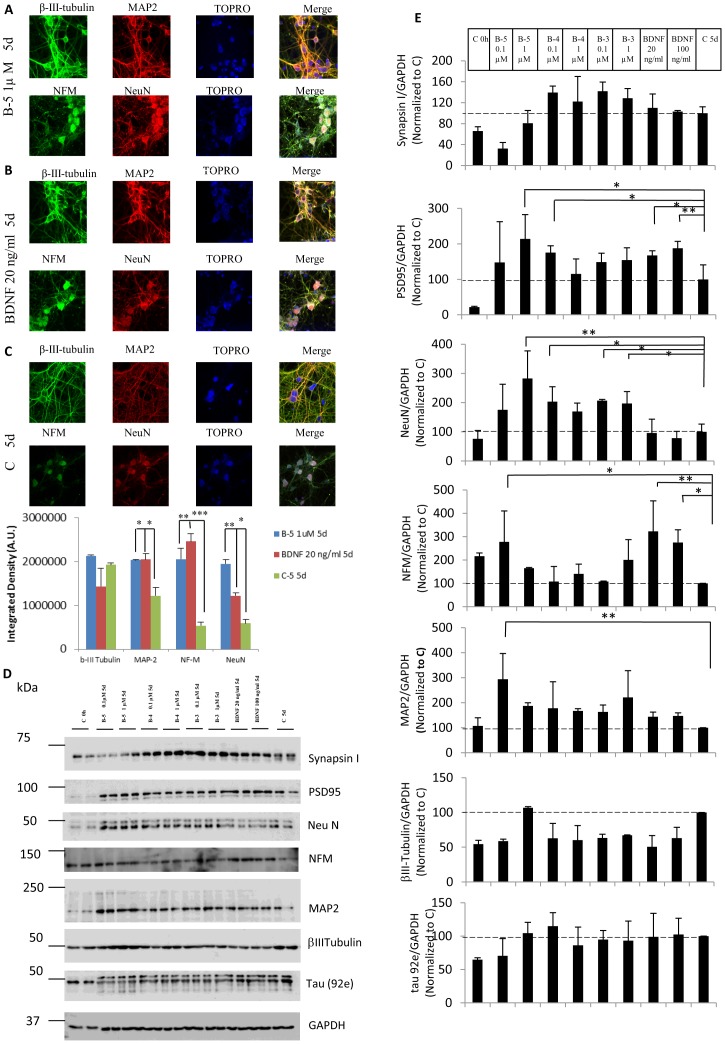
BDNF peptides B-5, B-4, and B-3 are neurogenic and neurotrophic. (A–C) Peptide B-5 induces the expression of the neuronal markers β-III-tubulin, MAP2, NFM, and NeuN in E18 primary hippocampal cell cultures. Representative confocal images illustrating double immunolabeling of β–III-tubulin (green) and MAP2 (red) and NFM (green) and NeuN (red) in cells treated for 5 days with (A) Peptide B-5 at 1 µM, (B) with BDNF, 20 ng/ml (0.79 nM) for 5 days, and,(C) vehicle only (control, C). TOPRO-3 (blue) was used to stain the nuclei. Magnification bar = 10 µm. The bottom panel shows the level of fluorescence intensity using Image J software to measure the level of expression. (D, E) BDNF peptides (B-5, B-4, and B-3) induce the expression of neuronal markers in E18 primary hippocampal neurons. (D) Representative Western blots showing an increase in the expression of the neuronal markers PSD95, NeuN, NFM, and MAP-2 in cells treated with the peptides (B5, B-4 and B-3) at concentrations of 0.1 µM and 1 µM or BDNF at concentrations of 20 or 100 ng/ml (0.79 or 3.95 nM respectively). GPADH was used as a loading control. (E) Quantification of the Western blots of neuronal markers shown in D. The integrated density value of the bands in Western blots was determined using densitometry (Fuji software, Multi Gauge, Version 3.0), and data was normalized to GAPDH and to control (medium treated cells for 5 days, C 5d). Data are shown as mean ± standard deviation, n = 3. 10% SDS-PAGE gels. *p<0.05, **p<0.01, ***p<0.001. One-way ANOVA/post-hoc test/Student’s *t* test.

Immunocytochemical studies revealed that peptide B-5 at a concentration of 1 µM induced an increase in the level of expression of MAP2, β-III-tubulin, NFM, and NeuN in mouse E18 hippocampal neurons after 5 days of treatment (Fig. 3A), as compared to vehicle-treated cells (Fig. 3C). The increase in the fluorescence intensity level of MAP2, NFM and NeuN staining with peptide B-5 was similar to the one obtained when cells were treated with BDNF, 20 ng/ml, (0.79 nM) (Fig. 3B), suggesting that the peptides could mimic at least in part the effects of the parent growth factor (BDNF). The fact that the cells treated with Peptide B-5 were NeuN-positive could mean that these cells were terminally differentiated and for that reason they could also be functional, since they were also positive for other neuronal markers, such as β–III-tubulin and MAP2. Peptide B-3 had similar effects on the expression of the neuronal markers (data not shown). To further elaborate the increase in the level of expression of the neuronal markers, we obtained the rainbow scale images corresponding to the confocal images in Figure 3 (Fig. S2). In the rainbow images, the warm colors (like red and yellow) represent the higher level of expression of the neuronal marker analyzed, and the cold colors (like blue) represent the lowest level of expression of the marker. White represents the highest level of expression whereas black represents no expression at all.

To biochemically study the effect of BDNF peptides in inducing expression of neuronal markers, protein samples were recovered from cells treated with peptides B-5, B-4, B-3, or vehicle for 5 days. As a positive control, protein samples were collected from cells treated with BDNF at 20 ng/ml (0.79 nM) or 100 ng/ml (3.95 nM) (Fig. 3D, E). Western blots of the cell lysates were developed with antibodies to Synapsin I, PSD95, NeuN, NFM, MAP2, β–III-tubulin, and tau 92e. The densitometric analysis showed that peptides B-5 and B-4 induced a significant increment in the expression of the postsynaptic marker PSD95 (*p* = 0.03, ANOVA for B-5 1 µM and *p* = 0.024, ANOVA for B-4 0.1 µM), similar to the increase obtained by the treatment with BDNF (at 20 and 100 ng/ml, *p* = 0.039, ANOVA and *p* = 0.012, ANOVA, respectively) (Figure 3E). Peptides B-5 (1 µM; *p* = 0.002, ANOVA), B-4 (0.1 µM; *p* = 0.043, ANOVA) and B-3 (0.1 µM; *p* = 0.038, ANOVA and 1 µM; *p* = 0.05, ANOVA) increased the expression of NeuN. Peptide B-5 (0.1 µM; *p* = 0.032, ANOVA) increased NFM expression in a similar way to the treatment with BDNF (20 ng/ml and 100 ng/ml; *p* = 0.011 and *p* = 0.034, ANOVA, respectively). MAP2 expression increased significantly with B-5 (0.1 µM; *p* = 0.009, ANOVA) treatment. Only peptide B-5 (1 µM) induced an increase in the levels of β–III-tubulin as compared to vehicle-treated cells for 5 days, but this increase was not significant. Neither BNDF nor peptides B-3, B-4 or B-5 had any significant effect on the level of tau (detected with 92e antibody) (Fig. 3D, E).

### Peptide B-3 Potentiates the Neuroprotective Effect of BDNF against H_2_O_2_-induced Toxicity

**Figure 4:**
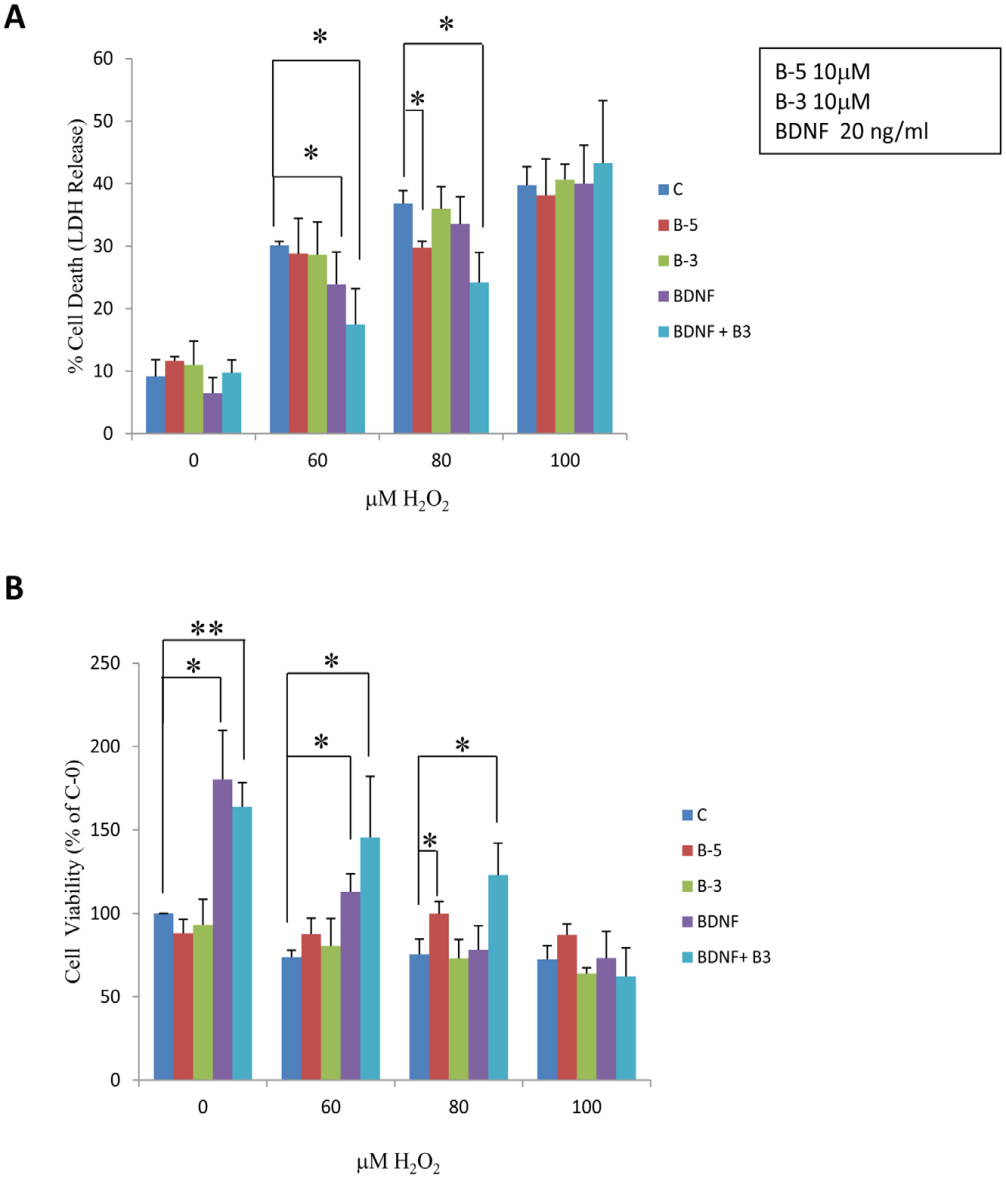
BDNF peptides potentiate the effect of BDNF in rescuing H_2_O_2_-induced neurotoxicity. (A) LDH cytotoxicity assay showing the percentage of cell death in hippocampal cells treated with increasing concentrations of H_2_O_2_ i.e. 0, 60, 80, and 100 µM for 6 h, and then after changing the medium, exposed to B-5, B-3, BDNF or BDNF+B-3 for 24 h. Peptide B-5 significantly reduced cell death caused by 80 µM H_2_O_2_ and Peptide B-3 significantly potentiated the neuroprotective effect of BDNF. (B) Percent viability of hippocampal cells by LDH assay. The cells were treated in the same way as in A. Peptide B-3 significantly increased the viability when combined with BDNF in cells not treated or treated with 60 or 80 µM of H_2_O_2_. Data were normalized to control (vehicle treated cells). * *p*<0.05, **p<0.01. ANOVA and/or Student’s *t* test, n = 3.

In order to evaluate the potential neuroprotective effect of BDNF peptides, we challenged the primary E18 hippocampal cells with 0, 60, 80 and 100 µM H_2_O_2_ for 6 h, and then cells were washed with culture medium and fresh culture medium was added containing the peptides B-5 or B-3 or BDNF or BDNF plus B-3 (Fig. 4 A, B). After 24 h, the cell viability was assayed by the LDH method. Cells treated with H_2_O_2_ showed a significant increase in cell death (ANOVA, *p*<0.0001) (Fig. 4A) with a concomitant reduction in cell viability (Fig. 4B) (ANOVA, *p*<0.001). The cells treated with BDNF after being exposed to H_2_O_2_ showed some reduction in cell death when compared to control medium treated cells, however, this was not statistically significant for most except for BDNF with 60 µM H_2_O_2_ treatment (0 µM H_2_O_2,_ ANOVA, *p* = 0.9377, Student’s *t* test, *p* = 0.2700; 60 µM H_2_O_2_, ANOVA, *p* = 0.0304,Student’s *t* test, *p* = 0.1076; 80 µM H_2_O_2_, ANOVA, *p* = 0.3699, Student’s *t* test, *p* = 0.3094; 100 µM H_2_O_2_, ANOVA, *p* = 0.3764, Student’s *t* test, *p* = 0.9519). Conversely, BDNF increased cell viability significantly at 0 and 60 µM H_2_O_2_ but was not effective at high H_2_O_2_ concentrations (80 µM and 100 µM) (0 µM H_2_O_2,_ ANOVA, *p* = 0.3167, Student’s *t* test, *p* = 0.0124; 60 µM H_2_O_2_, ANOVA, *p* = 0.0675, Student’s *t* test, *p* = 0.1072; 80 µM H_2_O_2_, ANOVA, *p* = 0.5762, Student’s *t* test, *p* = 0.7943; 100 µM H_2_O_2_, ANOVA, *p* = 0.4162, Student’s *t* test, *p* = 0.9508). These effects were enhanced by the combination of BDNF with Peptide B-3 in cells treated with 0, 60, and 80 µM but not 100 µM H_2_O_2_ (Cell death, 0 µM H_2_O_2,_ ANOVA, *p* = 0.7534, Student’s *t* test, *p* = 0.7876; 60 µM H_2_O_2_, ANOVA, *p* = 0.0247, Student’s *t* test, *p* = 0.0256; 80 µM H_2_O_2_, ANOVA, *p* = 0.4880, Student’s *t* test, *p* = 0.0189; 100 µM H_2_O_2_, ANOVA, *p* = 0.1603, Student’s *t* test, *p* = 0.5894; Cell viability, 0 µM H_2_O_2,_ ANOVA, *p* = 0.6739, Student’s *t* test, *p* = 0.0074; 60 µM H_2_O_2_, ANOVA, *p* = 0.0129, Student’s *t* test, *p* = 0.0790; 80 µM H_2_O_2_, ANOVA, *p* = 0.1956, Student’s *t* test, *p* = 0.0573; 100 µM H_2_O_2_, ANOVA, *p* = 0.2682, Student’s *t* test, *p* = 0.4745). These results suggest that peptide B-3 potentiates the neuroprotective effect of BDNF but alone is not sufficient to exert a significant effect. Peptide B-5 alone had a moderate effect in reducing the percentage of cell death when the hippocampal cells were treated with 80 µM of H_2_O_2_ (0 µM H_2_O_2,_ ANOVA, *p* = 0.1119, Student’s *t* test, *p* = 0.1911; 60 µM H_2_O_2_, ANOVA, *p* = 0.2057, Student’s *t* test, *p* = 0.7086; 80 µM H_2_O_2_, ANOVA, *p* = 0.4007, Student’s *t* test, *p* = 0.051; 100 µM H_2_O_2_, ANOVA, *p* = 0.4124, Student’s *t* test, *p* = 0.6888); also it significantly increased the cell viability with 60 and 80 µM of H_2_O_2_ (0 µM H_2_O_2,_ ANOVA, *p* = 0.6015, Student’s *t* test, *p* = 0.2478; 60 µM H_2_O_2_, ANOVA, *p* = 0.3191, Student’s *t* test, *p* = 0.084; 80 µM H_2_O_2_, ANOVA, *p* = 0.7923, Student’s *t* test, *p* = 0.0229; 100 µM H_2_O_2_, ANOVA, *p* = 0.7709, Student’s *t* test, *p* = 0.0717). We did not observe any potentiation of the neuroprotective effect of BDNF when used in combination with peptide B-5 (data not shown).

The confounding effect of endogenous BDNF cannot be ruled out from the above results. As mentioned before, hippocampal cells secrete small amounts of endogenous BDNF (17, 18). Nevertheless, in the absence of peptides, endogenous levels of BDNF were not sufficient to exert any protective effect in cells treated with H_2_O_2_.

### Peptides B-5 and B-3 Increase BDNF Expression

**Figure 5:**
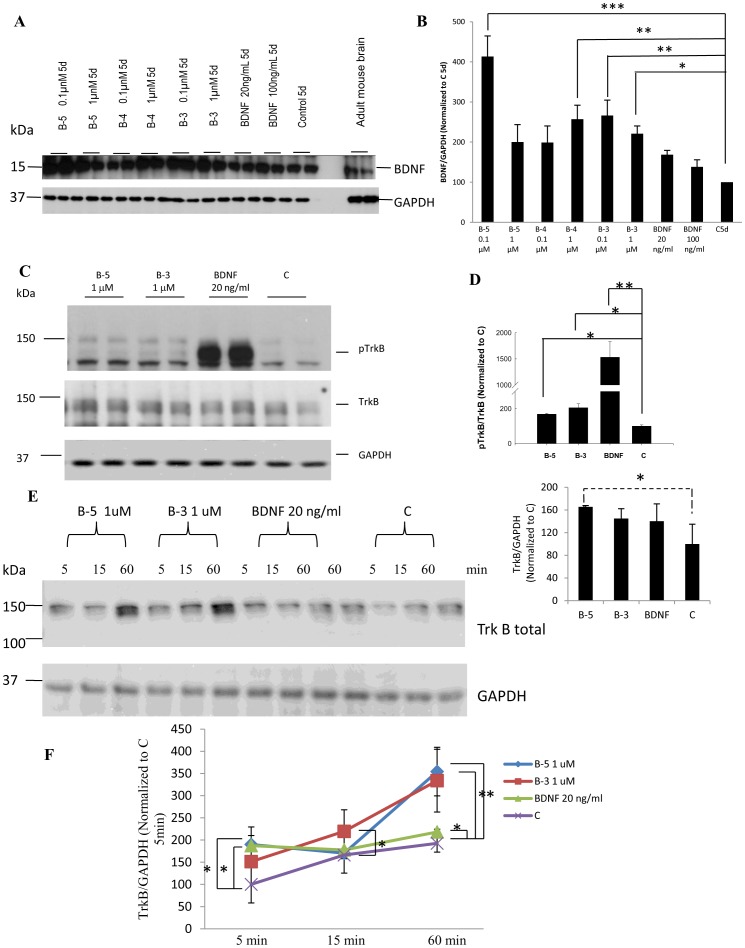
(A) BDNF peptides B-5, B-4 and B-3 induce the expression of BDNF. Western blot analyses of cells treated with peptides B-5, B-4 and B-3, or with BDNF, or vehicle for 5 days, showed an increase in BDNF expression in cells treated with the peptides. A sample of adult mouse brain was included as a control for the migration of the bands corresponding to pro-BDNF and BDNF. (B) Densitometric quantitation of the Western blots developed with anti-BDNF. Data was normalized to GAPDH as loading control and then to 5 days control vehicle treated cells, C 5d. (C) Peptides B-5 and B-3 activated TrkB receptor in primary E18 hippocampal cells. Western blots showing phosphorylation of TrkB at Tyr 706 on treatment with Peptide B-5 (1 µM), Peptide B-3 (1 µM) or BDNF (20 ng/ml, 0.79 nM) for 1 h as compared to control treated cells, C. Lower blots show the levels of TrkB receptor and the levels of GAPDH as a loading control. (D) Densitometric analysis of the Western-blots for pTrkB normalized to TrkB, and TrkB normalized to GAPDH. Control was taken as 100 percent in each case. (E) BDNF peptides (B-5 and B-3) increased the expression of TrkB. Increase in expression of TrkB in TrkB stably-expressing NIH-3T3 fibroblast cells, as a function of time. Cells were treated for 5, 15 or 60 min with B-5 (1 µM), B-3 (1 µM), BDNF (20 ng/ml), or vehicle only (Control, C). Western blots of total TrkB, and GAPDH included as a loading control. (F) Densitometric quantitation of the Western blots for TrkB normalized to GAPDH. Data are shown as mean ± standard deviation, n = 3. *p<0.05, **p<0.01, ***p<0.001. One-way ANOVA/post-hoc test/Student’s *t* test. Dashed line in (D) denotes that B-5 induction of TrkB expression almost approaches significance (*p* = 0.057, one-way ANOVA).

To investigate the molecular mechanism by which the BDNF peptides promoted neurogenic/neurotrophic activities, we treated the hippocampal primary cultured neurons with the peptides or BDNF for 5 days and compared with the control medium treated cells. Peptides B-5, B-4 and B-3 induced the expression of BDNF, probably potentiating its signaling (Fig. 5A, B). The strongest induction of the expression of BDNF was produced by Peptide B-5 at a concentration of 0.1 µM (ANOVA, *p*<0.001); at 1 µM this effect of Peptide B-5 was lost. Peptide B-4 at a concentration of 1 µM and peptide B-3 at both 1 µM and 0.1 µM induced a significant increase in the expression of BDNF (ANOVA, *p* = 0.027).

### Peptides B-5 and B-3 Increase Expression and Activation of TrkB in Hippocampal Primary Neuronal Cultures

Towards further understanding the feedback mechanism of action of the BDNF peptides, we studied the effect of 1 h treatment by these two peptides on the level and activation of TrkB receptor in the hippocampal primary cultured neurons. Peptides B-5 and B-3 induced a weak activation of TrkB phosphorylation in comparison to the activation of this receptor by BDNF in primary E18 hippocampal cells (Fig. 5C, D). A protein band at 145 kDa corresponding to the phosphorylation of TrkB receptor at tyrosine 706 which is one of the sites that gets rapidly phosphorylated on exposure of the ligand of this receptor for 1 h was observed. The level of total TrkB receptor and the level of GAPDH as a loading control were used as references. The normalization of the phosphorylated TrkB with total TrkB showed a strong and significant activation of TrkB by BDNF (ANOVA, *p*<0.001) as expected, and a relatively weak activation of the receptor by B-5 and B-3 (ANOVA, *p* = 0.035 and *p* = 0.0237 respectively). The fact that BDNF peptides are able to activate weakly the TrkB receptor suggests that they could act as partial agonists. The TrkB levels were increased on treatment with B-5 and B-3, almost reaching the significance level with B-5 (ANOVA, *p* = 0.057). BDNF also increased TrkB expression, however, this did not reach level of significance (ANOVA, *p* = 0.092) (Fig. 5C, D).

### BDNF Peptides Increase the Expression of TrkB Receptor in Fibroblast Cell Line

The rationale for using heterologous non-neuronal fibroblasts cells, NIH-3T3, stably expressing Trk receptors was to evaluate if the BDNF peptides were able to activate Trk (induce the tyrosine phosphorylation) in an heterologous environment in the absence of other neural-specific factors (23). In addition, NIH-3T3–TrkB cells are a well-established in vitro system to evaluate candidate ligands of TrkB receptors (21). The NIH 3T3 fibroblasts stably expressing the TrkB receptor were vehicle-treated or treated with peptides B-5 and B-3 at a concentration of 1 µM or with BDNF at a concentration of 20 ng/ml (0.79 nM) in medium without serum (to avoid the presence of endogenous BDNF from the serum) for 5, 15, or 60 min (Fig. 5E, F). In the time course experiment, both peptides B-5 and B-3 induced a marked increase in the expression of TrkB after 60 minutes of treatment (ANOVA, *p* = 0.041 and *p* = 0.0392 respectively) (Fig. 5C, D). BDNF increased TrkB expression only after initial 5 minutes (*p* = 0.029); the effect did not persist at 15 and 60 minutes. These data support the findings shown above in Figures 5C, D, where an increase in TrkB receptor in E18 primary hippocampal cells, after treatment with Peptide B-5 and B-3 is documented. Peptides B-5 and B-3 probably potentiate the BDNF signaling by inducing an increase in the expression of TrkB. The neurotrophic effect of these peptides could be mediated by inducing increase in the expressions of both BDNF (Figure 5A, B) and TrkB (Fig. 5C–F).

### Activation of the TrkB Receptor by the BDNF Peptides can be Blocked by the TrkB Inhibitor K252a

**Figure 6:**
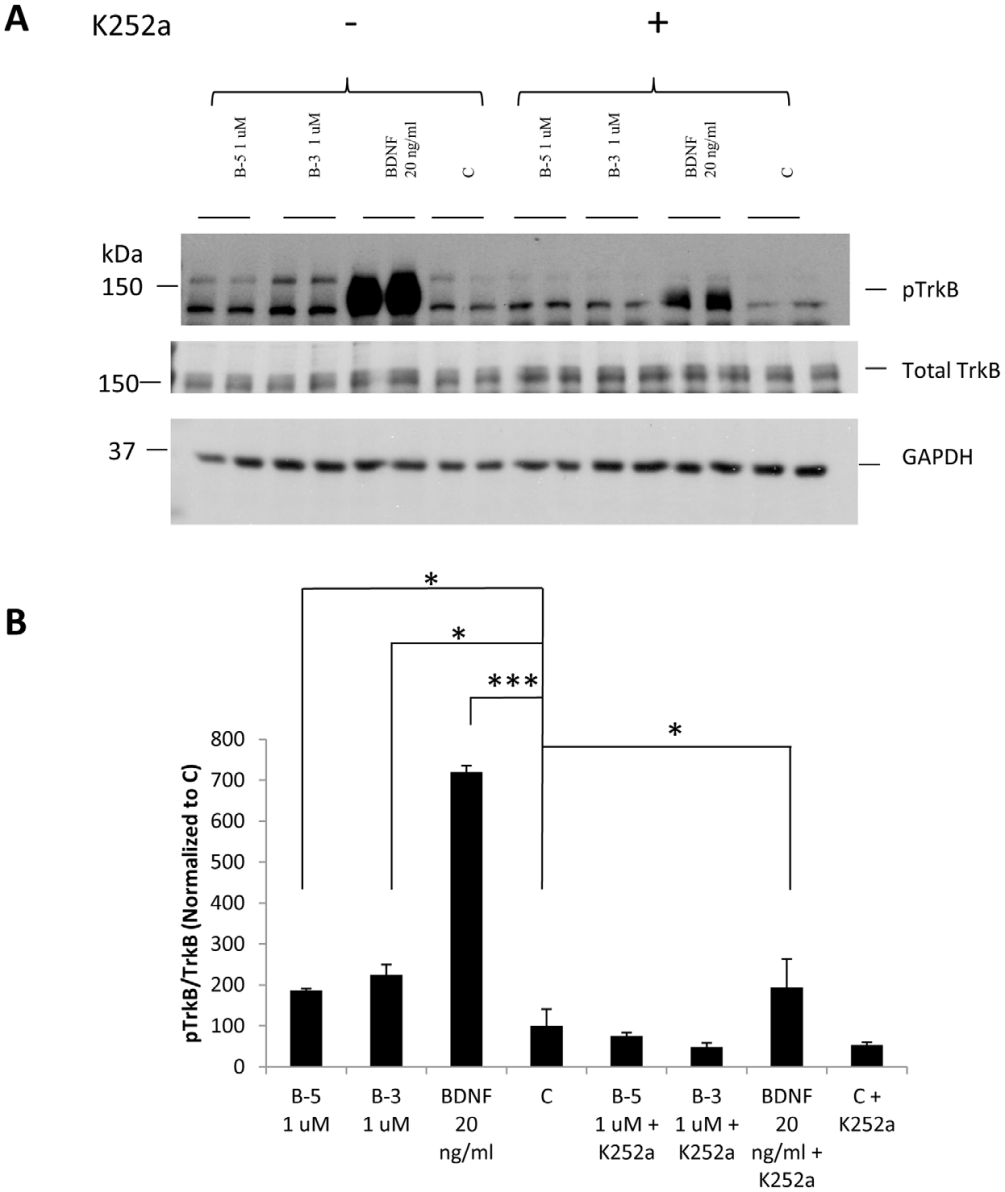
Activation of the TrkB receptor by BDNF peptides B-5 and B-3 can be blocked by the TrkB inhibitor, K252a. (A) Cells were pretreated with or without K252a for 1 h and then exposed to Peptide B-5 or Peptide B-3 at 1 µM or 20 ng/ml BDNF for 5 min. Western blots of pTrkB (Tyr 706), total TrkB and GAPDH included as loading control. (B) Densitometric quantitation of the Western-blots for pTrkB normalized to TrkB. Data are shown as mean ± standard deviation, n = 3. *p<0.05,**p<0.01, ***p<0.001. One-way ANOVA/post-hoc test/Student’s *t*-test.

To further confirm the activation of the TrkB receptor by the peptides, we pretreated mouse primary E18 hippocampal cells with the Trk family inhibitor K252a for 1 h, and then added the growth factor BDNF (20 ng/ml) or the peptides B-5 or B-3 (at 1 µM) for 5 min and compared to vehicle-treated cells used as a control (C). By Western blots, we found a clear inhibition of the phosphorylation of TrkB at Tyrosine 706 by K252a (Fig. 6A, B). The increase in the phosphorylation of the TrkB receptor was significant for the treatment with B-5 and B-3 and BDNF (B-5, 1 µM, ANOVA, *p* = 0.0341; B-3, 1 µM, ANOVA, *p* = 0.019; BDNF, 20 ng/ml, ANOVA, *p* = 0.0003), but when cells were pretreated with K252a, there was a dramatic reduction in the activation of this receptor. However, BDNF, 20 ng/ml still induced a significant activation of TrkB (ANOVA, *p* = 0.032). These results confirmed that the peptides activated the TrkB receptor, and that this activation can be blocked by the Trk inhibitor, K252a. Total TrkB is shown as a reference, but we did not see a significant change in its expression. Since the treatment with the peptides was for a very short time (5 min), changes in the level of TrkB expression were not expected. There was an apparent specificity of the peptides B-5 and B-3 for activating TrkB receptor since we tested its activity on NIH 3T3 cells stably expressing TrkC, and neither the peptides nor BDNF were able to activate this receptor, which is normally activated by its ligand NT-3 (Fig. S3).

### Peptides B-5 and B-3 Work as Partial Agonists and as Partial Antagonists of BDNF in TrkB-stably Transfected Cells

**Figure 7:**
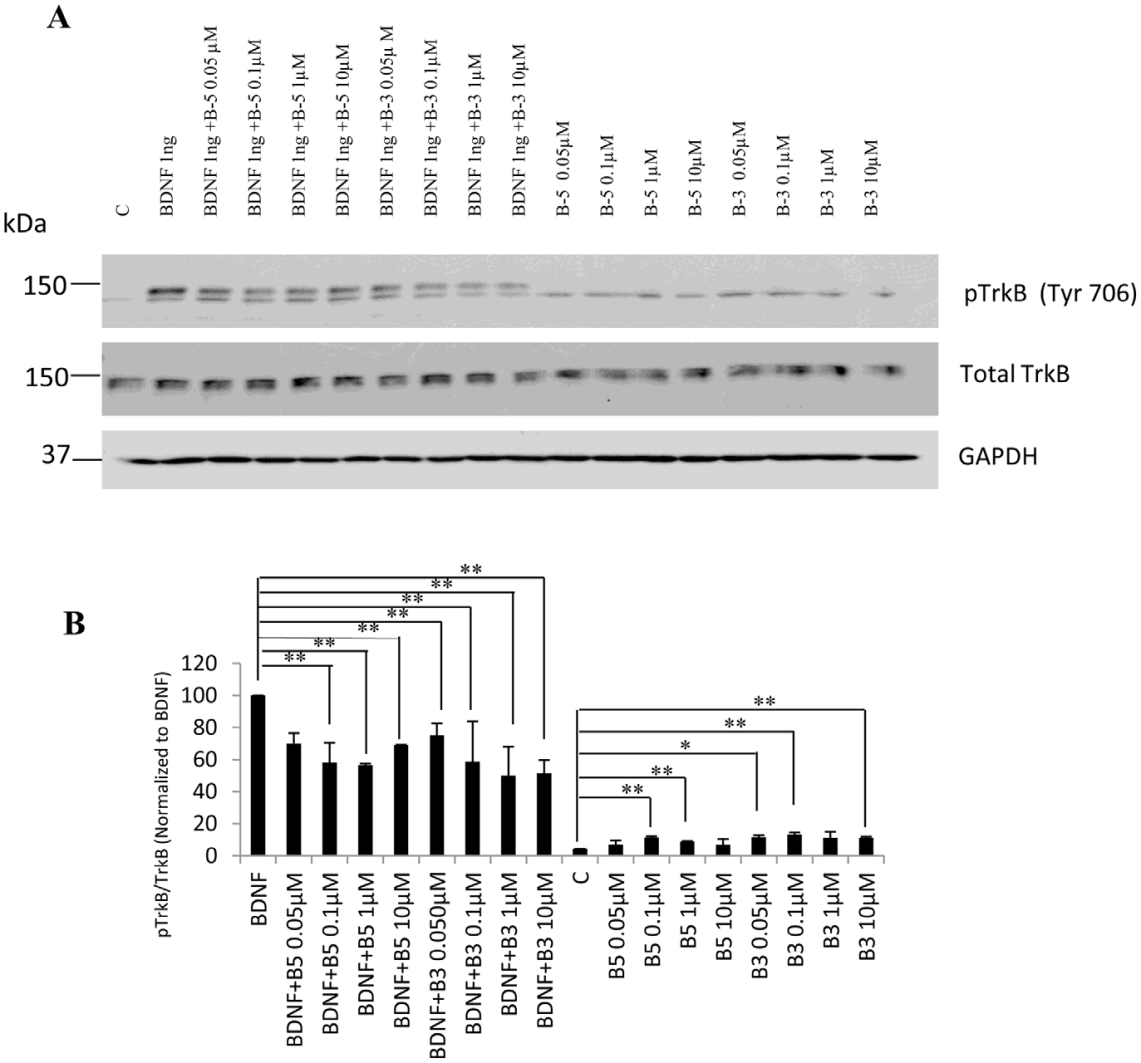
(A, B) BDNF peptides act as partial agonists and antagonists of BDNF. Competition experiments showing inhibition of the activation (pTrkB) of the TrkB receptor when the TrkB receptor stably expressing NIH-3T3 fibroblasts were treated with increasing concentrations of peptides B-5 or B-3 in the presence or absence of BDNF for 15 min. (A) Western blots of pTrkB (Tyr 706) and of total TrkB. GAPDH is included as a loading control. (B) Densitometric quantitation of Western blots for pTrkB normalized to TrkB (after normalizing TrkB to GAPDH) and shown as a percentage of BDNF. Data are shown as mean ± standard deviation, n = 3. *p<0.05, **p<0.01. One-way ANOVA/post-hoc test/Student’s *t* test.

To evaluate a possible competitive role of the peptides in the activation of TrkB receptor by BDNF, we used a fibroblast (NIH 3T3) cell line stably expressing the TrkB receptor. Cells were vehicle-treated or treated with BDNF 1 ng/ml (0.04 nM) in the presence or absence of 0.05 µM to 10 µM B-5 or B-3 for 15 min (Fig. 7A). Both B-5 and B-3 showed a significant competitive inhibition (post-hoc tests, *p*<0.01) of the activation of TrkB receptor by BDNF, and the effect was dose-dependent, suggesting a role of the peptides as partial antagonists of the BDNF pathway since they competed for the activation of the TrkB receptor but they did not block completely its activation by BDNF (Fig. 7A, B). As observed in primary neuronal cultures (shown in Fig. 5C, D), the treatment with the peptides alone for 15 min induced a weak but significant activation of TrkB receptor when compared to control (ANOVA, *p*<0.01).

### Protein Synthesis Inhibitor Cycloheximide and TrkB Inhibitor K252a Blocked the Increase in the Expression of BDNF and TrkB Produced by the Peptide B-5 or by BDNF, in Mouse E18 Primary Hippocampal Cells during 2 Days of Treatment

**Figure 8:**
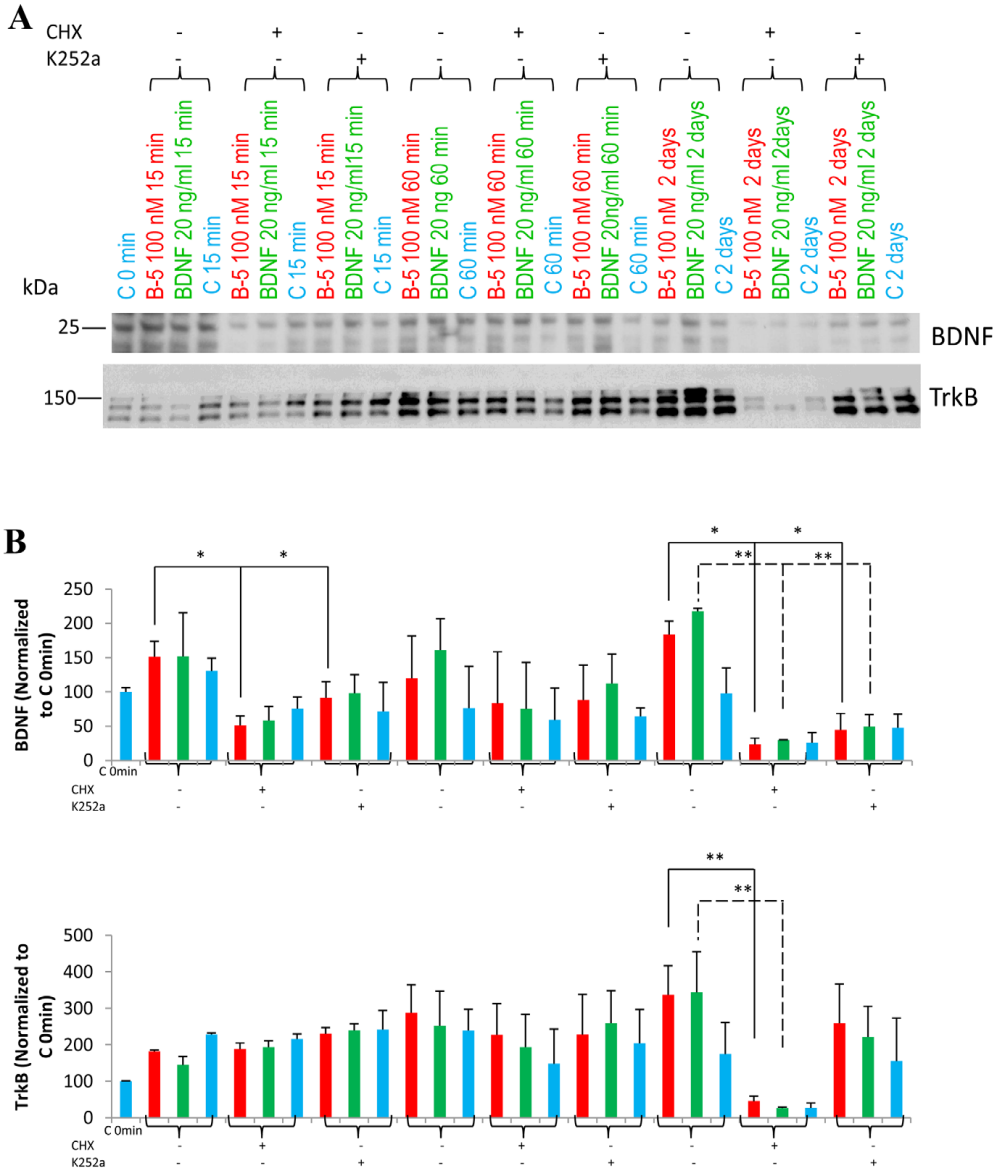
(A,B) The increase in expression of BDNF in primary hippocampal cells caused by BDNF peptides or BDNF after two days of treatment can be blocked by pre-exposure (1 h before adding the peptides) to protein synthesis inhibitor, cycloheximide (CHX) or to TrkB inhibitor, K252a. (A) Western blots of BDNF, and total TrkB, of hippocampal cells treated with 0.1 µM peptide B-5 or 20 ng/ml BDNF or vehicle treated, and when indicated, also pretreated with CHX or K252a (B) Densitometric quantitation of the Western-blots for BDNF and TrkB normalized to the corresponding control cells (control, C) in each condition. Data are shown as mean ± standard deviation, n = 3. *p<0.05, **p<0.01. One-way ANOVA/post-hoc test/Student’s *t* test.

To evaluate whether the effect of B-5 and B-3 in induction of the expression of BDNF and TrkB was via the activation of the TrkB receptor and required signal transduction via TrkB and new protein synthesis, we pre-treated mouse embryonic E18 cultured hippocampal cells with the protein synthesis inhibitor, cycloheximide (CHX) or the Trk inhibitor, K252a for 1 h, and then added Peptide B-5 at a concentration of 0.1 µM (the dose that gave the maximum induction of BDNF expression, Fig. 5A,B) or BDNF 20 ng/ml as a positive control (Fig. 8A, B). We studied a time course of these treatments with Peptide B-5 or BDNF for 15 min, 60 min and 2 days. At 2 days there was a significant (ANOVA, *p*<0.05) inhibition of the increase in the expression of BDNF and of TrkB by the Peptide B-5 in the presence of CHX; and there was no more significant induction of the expression of either BDNF or TrkB by B-5 in cells treated with the Trk inhibitor (K252a) (Fig. 8A, B). The trends in the reduction of BDNF expression were similar to 2days at 15 min and 60 min time points, however, TrkB inhibition was evident only at 60 minute time point, and reached a marked significance at 2 days time point. Since the effect of BDNF peptides was not completely blocked by K252a, there is a possibility that these peptides activate alternate pathways that lead to expression of BDNF and TrkB.

## Discussion

Therapeutic modulation of BDNF level remains a promising treatment strategy for neurological and psychiatric disorders in which the level of BDNF is dysregulated. However, the low plasma stability and low BBB permeability because of its moderately large size and ionic charge practically precludes the use of this neurotrophic factor as such for therapeutic usage, at least via peripheral administration. For instance, in a phase III clinical trial for the treatment of amyotrophic lateral sclerosis (ALS), a daily subcutaneous administration of BDNF offered no clinical benefit [26]. Alternatively, direct administration of BDNF into the CNS to achieve beneficial neurotrophic effects is a promising approach [27]–[29], however, there are also some considerations with this strategy that need to be taken into account. The CNS is composed of extremely delicate neural tissue sustained in a tightly controlled homeostatic environment, and direct intraventricular or intrathecal administration of a growth factor can cause undesirable effects. Direct administration of BDNF into the CNS has been reported to cause weight loss, dysaesthesias (impairment of sensation), and in some cases, pain [27]–[29]. Direct administration into CNS can be a better alternative if effective concentrations of the neurotrophic factor can be achieved at precise sites of degenerating neurons, while limiting the spread to distant sites to avoid undesirable effects. One method to attain this can be gene delivery via adeno-associated viral vectors (AAVs) [30], [31]. However, this approach is now in evaluation, and it requires additional improvements to guarantee the safety of the patients [32], [33]. Other alternatives include non-pharmacologic approaches for BDNF augmentation such as exercise and diet modulation. Physical exercise increases BDNF level in the hippocampus and the cortex, and may enhance learning and memory, synaptic plasticity, and neurogenesis [34]. Caloric restriction also affects the level of BDNF [35]. However, changes in BDNF expression level due to exercise or caloric restriction are low compared with the direct administration of the neurotrophic factor by infusion. Epigenetic modulation of gene transcription, as an alternative approach, can be achieved through direct methylation of DNA or by post-translational modification of histones, which can either repress or promote gene transcription. Fear conditioning has been shown to differentially regulate the expression of BDNF mRNAs, following BDNF DNA methylation [36], [37]. Drugs that are able to increase BDNF level in the brain include antidepressants [38] e.g. lithium increases BDNF concentrations in serum by 30% [39], and ampakines increase BDNF and improve stabilization of LTP and long-term memory in a mouse model of Huntington’s disease [40]. Whether these drugs induce sufficient changes in BDNF level to be useful for human diseases remains to be determined. Also remaining to be evaluated are the mechanisms that these drugs employ to modulate BDNF expression, since most of them can also activate alternative cellular signaling pathways, generating a complex mechanism of action.

For the above mentioned reasons, the development of small molecules that mimic the effects of BDNF, and depict enhanced permeability and stability can be very useful in the process of generating new drugs. The development of peptide mimetics of BDNF allows simple and controlled modulation of neurotrophic factor activities. In this context, the present study describes the initial screening of synthesized BDNF peptides and the evaluation of their capabilities to modulate the BDNF function in an in-vitro model. Our results demonstrate that the BDNF tetrapeptides, in particular B-5 and B-3, corresponding to active regions of BDNF, are neurogenic and neurotrophic, and can modulate BDNF activity in a partial agonistic/antagonistic way, and by enhancing the expression of BDNF and TrkB. The increase in the expression of BDNF suggests a feedback mechanism, and it could be due to an increase in BDNF secretion, which implies that the peptides could be acting as secretagogues (molecules that induce the secretion of other molecules). However, this remains to be further evaluated; especially considering the fact that hippocampal neurons make and secrete significant amounts of endogenous BNDF [18]. This need to be taken into consideration when evaluating the potential agonist/antagonist effects of the peptides; thus, the current results need to be interpreted in a prudent manner. There is a possibility that the peptides B-5 and B-3 mainly function as agonists; they work as antagonists only in the presence of elevated BDNF. The induced expression of BDNF by these peptides can have important effects in modulating the synaptic plasticity of the hippocampal neurons. The effect of the BDNF peptides in the induction of the activation of the receptor TrkB and in the induction of its expression were consistent in both the primary hippocampal cultures and in the cell line NIH-3T3- TrkB. Since the NIH-3T3- TrkB cell line is a non-neuronal heterologous system that lacks other neuronal-specific factors; we can expect that confounding effect of other endogenous neural factors involved in the activation of the TrkB receptor is least likely. In the case of the expression of TrkB in the cell line, it has to be considered that this modified cell line was generated by the transfection of the expression vector pJM8 (derived from the vector pMEX), that contains the cDNA of the rat trkB under the control of the MSV promoter (Mouse sarcome virus promoter) which is a different promoter from the original neuronal trkB promoter; consequently the mechanism of induction of trkB expression could be different between the neuronal cells and the genetically modified NIH-3T3 cell. We cannot rule out that the increase in expression of TrkB we observed in the experiments with the NIH 3T3 cells might be due to a decrease in its degradation when cells were treated with the BDNF peptides.

Given that these compounds are short peptide mimetic molecules of the neurotrophic factor, in most of the cases they should be able to cross the BBB when administered peripherally [19], [20], [41], [42]. Nonetheless, the BBB permeability of these peptides remains to be experimentally analyzed and constitutes one of the practical limitations of the current study. In contrast to the non-peptide origin of the small molecules previously used [2] for activating TrkB pathway, these compounds (B-1 to B-5) which also modulate this pathway, are tetrapeptides (derived from the active site of BDNF). These peptides compare favorably to the BDNF derived cyclic peptides generated previously [43].

Peptides investigated in the present study were found to be non-toxic to primary cultured hippocampal cells, and were able to induce the expression of neuronal markers. These findings suggest the potential therapeutic use of these peptides in neurodegenerative diseases such as AD and other cognitive disorders. In addition, B-3 showed neuroprotective effect against H_2_O_2_ induced toxicity in combination with BDNF. The fact that peptide B-3 and BDNF together show an additive effect on the survival of the cells exposed to H_2_O_2_ in comparison to the response elicited by BDNF or B-3 alone implies that B-3 could be acting through an alternative pathway besides the BDNF signaling pathway. This is in accordance with the proposed mechanisms of previously reported small molecules that mimetize partially the functions of BDNF [20], [43]–[45]. The activation of alternative signaling cascades by these BDNF peptides which emerges as a likely possibility based on our data remains to be evaluated in future studies. Of note, even though we found that peptide B-5 is a better enhancer of BDNF expression as compared to peptide B-3 in later experiments, we did not find any potentiation of neuroprotective effect of BDNF by peptide B-5 against H_2_O_2_ induced toxicity. This further strengthens the idea that these peptides lead to activation of multiple signaling cascades differentially. Alternatively, the confounding effect of endogenous BDNF on these results can’t be excluded completely.

Here, we demonstrate that the BDNF peptides can function both as partial agonists and partial antagonists. They act as agonists when they synergize with BDNF to protect the cells against oxidative stress, and they act as antagonists when they compete with BDNF to activate the TrkB receptor. A partial agonist is an agent that elicits a maximum response that is less than that of an agonist and acts as an antagonist in the presence of full agonist. While in the absence of a full agonist, partial agonists show functional agonist activity and bind to the receptor to produce a response [46]–[48]. Thus, it is possible that the peptides act differently depending on their concentration comparing to the level of the original ligand (BDNF) or depending on the conditions e.g. whether the cell is under stress or not. Evidence suggests that excess BDNF is involved in the pathogenesis of epilepsy, mania and autism. Pharmacologic agents that can decrease the BDNF-TrkB pathway signaling partially or to a certain extent, may be therapeutic for these diseases since blocking the BDNF-TrkB pathways with complete TrkB antagonists can lead to undesirable effects. A fine regulation of this pathway is thus warranted that can be achieved with the use of small molecules (like the BDNF peptides in this study) that can work both as partial agonist and antagonist leading to an optimal balance of this cascade.

In general, we looked for a small molecule that could mimic the neuroprotective effects of the complete molecule of the growth factor without causing adverse effects associated with the original ligand. It is likely that the BDNF peptides in the present study do not activate the pain related pathway associated to BDNF treatment [49], because these are small molecules that mimetize BDNF and they show activation of TrkB receptor as its principal pathway; the peptides did not activate NT3/NT4 receptor, TrkC. These peptides generated a moderate activation of the TrkB receptor as compared with the activation achieved with the complete growth factor, BDNF, at the time points evaluated, but it is possible that the peptides had a temporal kinetics different than that of BDNF, and it may take longer time to reach the maximum activation of the TrkB receptor, but this remains for further evaluation.

Up to the best of our knowledge, this is the first report of the role of BDNF in protection against oxidative stress caused by H_2_O_2_ in primary hippocampal neurons. Nonetheless, there are few reports which mention that BDNF could enhance survival of H_2_O_2_ stressed cells [50]. The prevention of oxidative stress and the reduction in reactive oxygen species (ROS) are considered to be promising approaches for neuroprotection in neurodegenerative diseases [51]. In the present study, as mentioned before, the combination of BDNF plus one of the BDNF peptides (B-3) was more potent in enhancing the survival of hippocampal cells previously exposed to H_2_O_2,_ probably acting in a partial agonistic way. Small molecules that mimic a particular ligand, like in the case of the peptides used in the present study, can bind to their receptors and disrupt protein-protein interactions inhibiting the functions they mediate, or they could act as activating ligands, though there may be differences from the natural ligand with respect to the coupling and kinetics of the induced signaling [21], [43]. The differential activation of downstream signaling by interaction with ligands and receptors may have an active involvement in the partial agonistic/antagonistic roles of the BDNF peptides used in this study.

**Figure 9:**
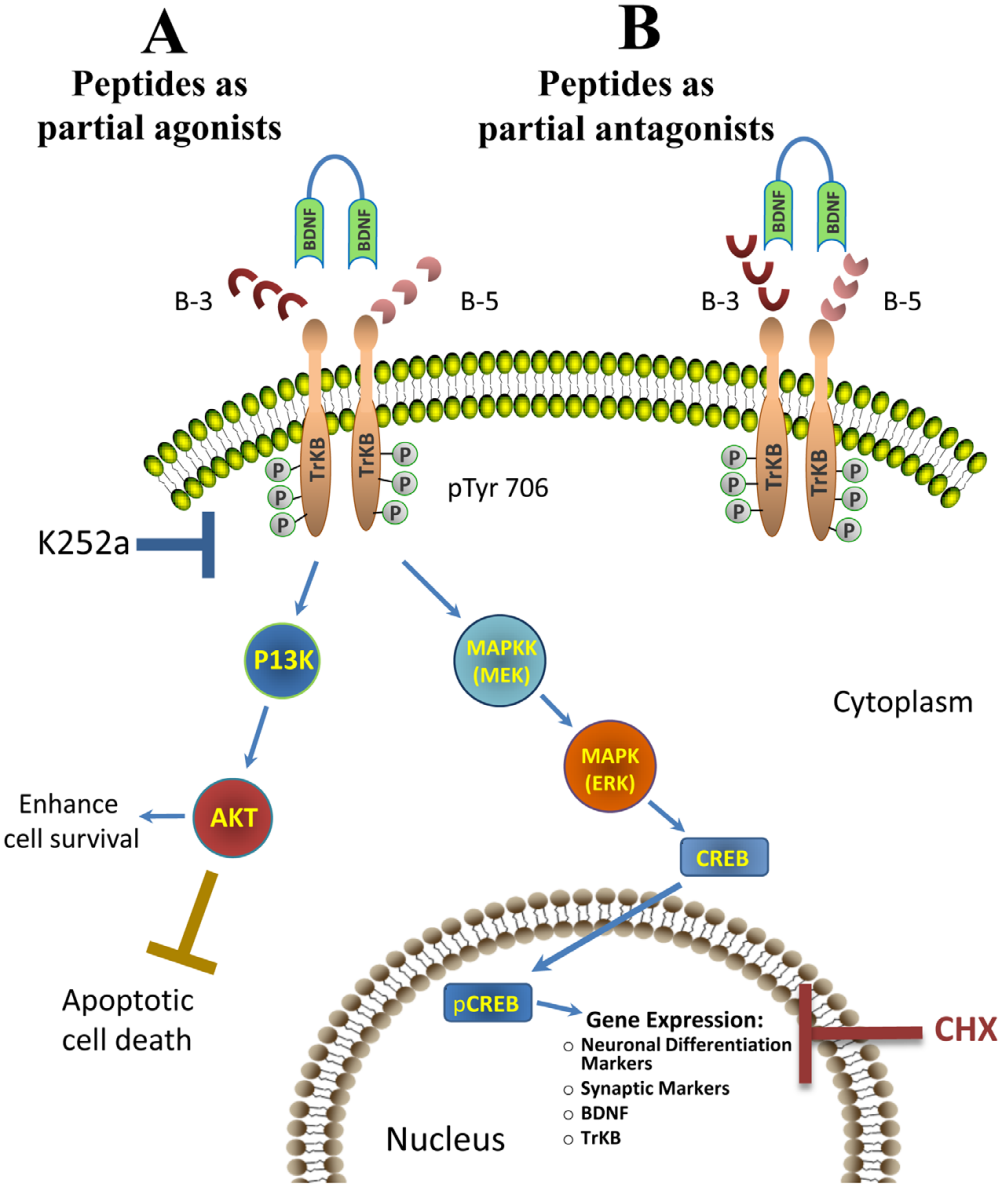
Proposed mechanism of action of BDNF peptides. BDNF peptides B-5 and B-3 may interact with or compete for the binding site of BDNF to its transmembrane receptor TrkB. Depending on the concentration or the cellular state or condition (i.e. during stress, like in the presence of H_2_O_2_), the peptides could act as partial agonists or partial antagonists. Fig. 8A shows the partial agonistic role of the peptides. In this case, the peptides favor the activation of the TrkB receptor, and in the presence of BDNF, they synergize with it. Once the TrkB receptor gets activated, it is dimerized and autophosphorylated (one of the residues that gets phosphorylated is the Tyr 706) and the signal is transduced. The cascades that could be activated by the peptides include the differentiation pathway through MAPK and pCREB regulating gene expression of markers of neuronal phenotype and plasticity, and also the expression of BDNF and TrkB, giving the possibility of a feedback mechanism. The other cascade that could be activated by the peptides is the survival one, in which PI3K and AKT participate to enhance survival and inhibit cell death. Fig. 8B represents the partial antagonistic role of the peptides where the peptides compete with BDNF for the activation of the receptor blocking the TrkB activation by BDNF and its signal transduction pathway. The sites where the TrkB inhibitor K252a and the protein synthesis inhibitor CHX can block the pathway are shown with a grey and red bar, respectively.

The proposed mechanism of action of the BDNF peptides, B-5 and B-3, is that they may interact or compete for the binding site of BDNF to its transmembrane receptor TrkB (Fig. 9). Depending on the concentration or the cellular state (for instance, during stress, like in the presence of H_2_O_2_), they could act as partial agonist or partial antagonists. Alternatively, they might also activate other receptors (which remain to be investigated). Once the TrkB receptor gets activated, it is dimerized and autophosphorylated (one of the residues that gets phosphorylated is the Tyr 706), afterwards, the signal is transduced, and two principal cascades can be activated, (i) the differentiation pathway through MAPK and pCREB, regulating gene expression of markers of neuronal phenotype and plasticity, and also the expression of BDNF and TrkB resulting in the possibility of a feedback mechanism; and (ii) the cascade involving PI3K and AKT that regulates survival and cell death. Alternatively, the BDNF pathway can activate the PLC-γ signaling that is involved in activity dependent plasticity. The increase in expression of BDNF and TrkB by Peptides B-5 and B-3 probably work like a feedback system. The inhibitor K252a blocks the activation of TrkB at the beginning of the pathway, and CHX blocks the protein synthesis at the end of the pathway (Fig. 9).

Brain derived neurotrophic factor has multiple effects in regulating neuronal function and survival, so it is an attractive molecule to target for developing new therapeutic approaches to neurological diseases. Nevertheless, as mentioned before, it is still a challenge to deliver this growth factor to the appropriate region of the CNS and to maintain its prolonged expression. For this reason, the peptides described in this study represent new tools for modulating the BDNF pathway and they could have therapeutic potential. Previous studies [52], [53] reported the use of small molecule BDNF mimetics that inhibit Aβ-induced neuritic dystrophy and neuronal death in hippocampal slice cultures, demonstrating the relevant potential role of molecular mimetics in the therapeutics of Alzheimer’s disease. Also, previously, we described ciliary neurotrophic factor (CNTF) derived peptide mimetics which showed beneficial effects on neurogenesis, synaptogenesis, synaptic plasticity, and cognition in mouse and rat models of Alzheimer’s disease [54]–[59].

Applications of small molecule mimetic drugs that target protein kinases involve not only neurological diseases, but also a variety of other disorders including obesity, metabolic syndrome, muscular degenerations, ulcerative lesions, diabetes, and cancer [42], [60]. In the case of cancer, it can be useful to find a molecule that works as an antagonist of Trk receptors, since these receptors by virtue of their roles in the regulation of growth, differentiation and programmed cell death are reported to be involved in oncogenesis [61].

In summary, the BDNF peptides described here which are only four amino acid long, are non-toxic, and exert neurogenic and neurotrophic effects in neuronal hippocampal cell culture, and, thus, could serve as lead compounds for the development of neurotrophic drugs with enhanced permeability and stability.

## Supporting Information

Figure S1 **Effect of BDNF peptides on hippocampal neural precursor cells (NPCs) from E18 mice.** (A) Peptides B-5, B-4 and B-3, were able to induce the expression of β-III-tubulin, synapsin I, and NeuN. Representative Western blot analyses of cells treated with peptides B-5, B-4, B-3, B-2, B-1 and BDNF or vehicle for 5 days. Peptides were used at three different concentrations, 0.1, 0.5 and 1 µM; BDNF was used at 20 ng/ml. (B) Densitometric quantification of the Western blots of β–III tubulin, synapsin I, and NeuN, normalized to GAPDH (as loading control).
(TIF)

Figure S2 **Rainbow images with scale, corresponding to the immunocytochemistry images shown in** **Figure 3** **.** The warm colors (like red and yellow) represent the higher level of expression of the neuronal marker analyzed, and the cold colors (like blue) represent the lowest level of expression of the marker. White represents the highest level of expression whereas black represents no expression at all. (A–C) Peptide B-5 and BDNF induce the increase in the level of expression of the neuronal markers β-III-tubulin, MAP2, NFM, and NeuN in E18 primary hippocampal cell cultures as compared to the non-treated control. Representative confocal images in the format of rainbow scale, illustrating double immunolabeling of β-III-tubulin and MAP2 or NFM and NeuN in cells treated for 5 days with Peptide B-5 at 1 µM (A), with BDNF at 20 ng/ml (0.79 nM) (B), and vehicle only (control) (C). TOPRO-3 (blue) was used to stain the nuclei. Magnification bar = 10 µm. To the left side of each image in rainbow format, rainbow color scale is shown.
(TIF)

Figure S3 **Neither BDNF (20 ng/mL) nor peptides B-5 (1** **µM) and B-3 (1** **µM) were able to modify the expression of TrkC in TrkC stably-expressing NIH-3T3 fibroblast cells.** (A) Western blots of anti-pan-phospho-Trk (Y490) and of total TrkC. GAPDH was used as a loading control. (B) Densitometric quantitation of the Western blots for pTrk normalized to total TrkC (after normalizing TrkC to GAPDH). Data are shown as mean ± standard deviation, n = 3.
(TIF)
