# Supplementary figures and images for: Neurogenic and Neurotrophic Effects of BDNF Peptides in Mouse Hippocampal Primary Neuronal Cell Cultures
Source: PLoS One. 2013 Jan 8;8(1):e53596. doi: 10.1371/journal.pone.0053596 (PMC3539976; doi:10.1371/journal.pone.0053596)

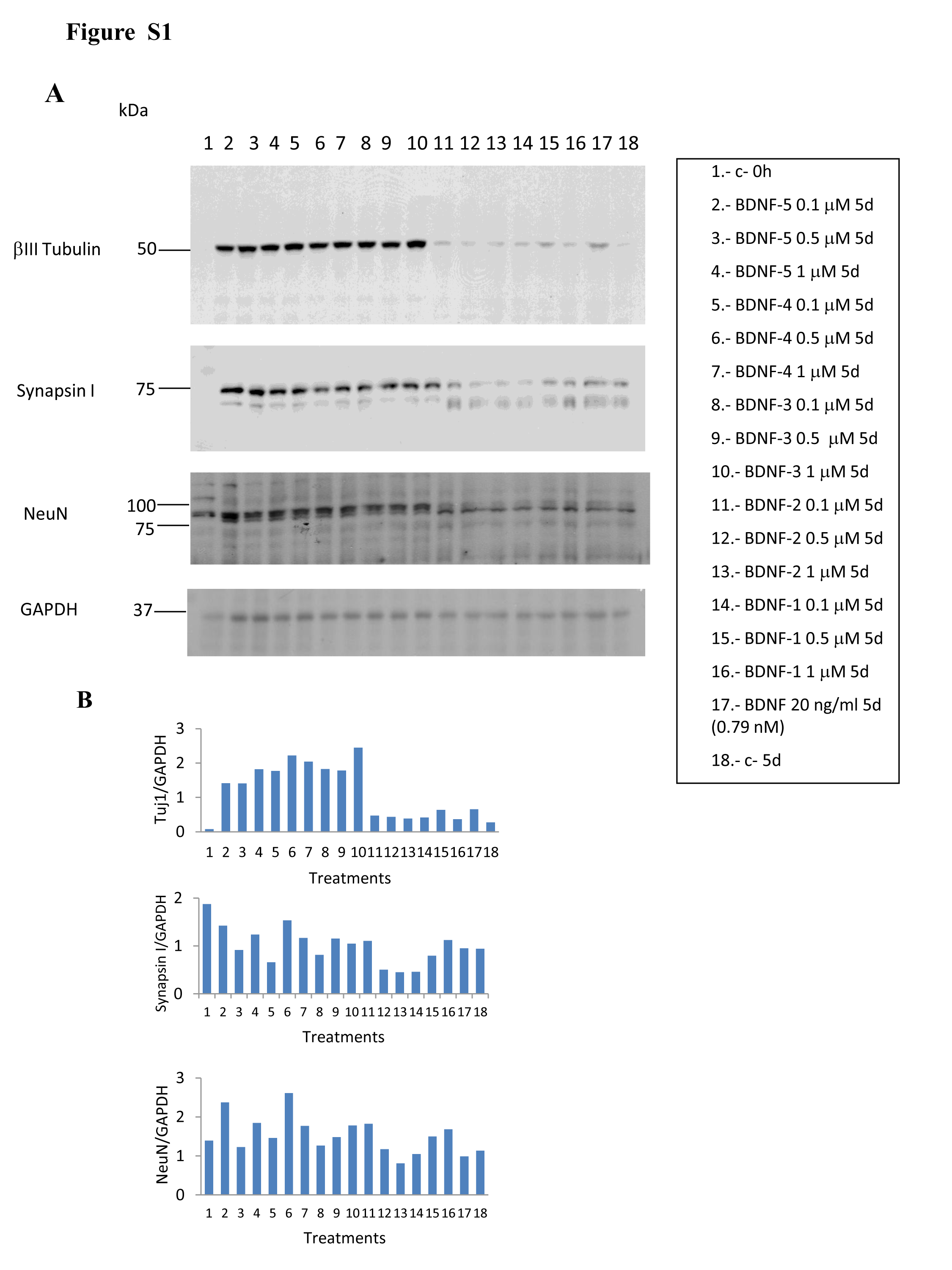

Supplement: Figure S1 — Effect of BDNF peptides on hippocampal neural precursor cells (NPCs) from E18 mice. (A) Peptides B-5, B-4 and B-3, were able to induce the expression of β-III-tubulin, synapsin I, and NeuN. Representative Western blot analyses of cells treated with peptides B-5, B-4, B-3, B-2, B-1 and BDNF or vehicle for 5 days. Peptides were used at three different concentrations, 0.1, 0.5 and 1 µM; BDNF was used at 20 ng/ml. (B) Densitometric quantification of the Western blots of β–III tubulin, synapsin I, and NeuN, normalized to GAPDH (as loading control). (TIF) [file pone.0053596.s001.tif]

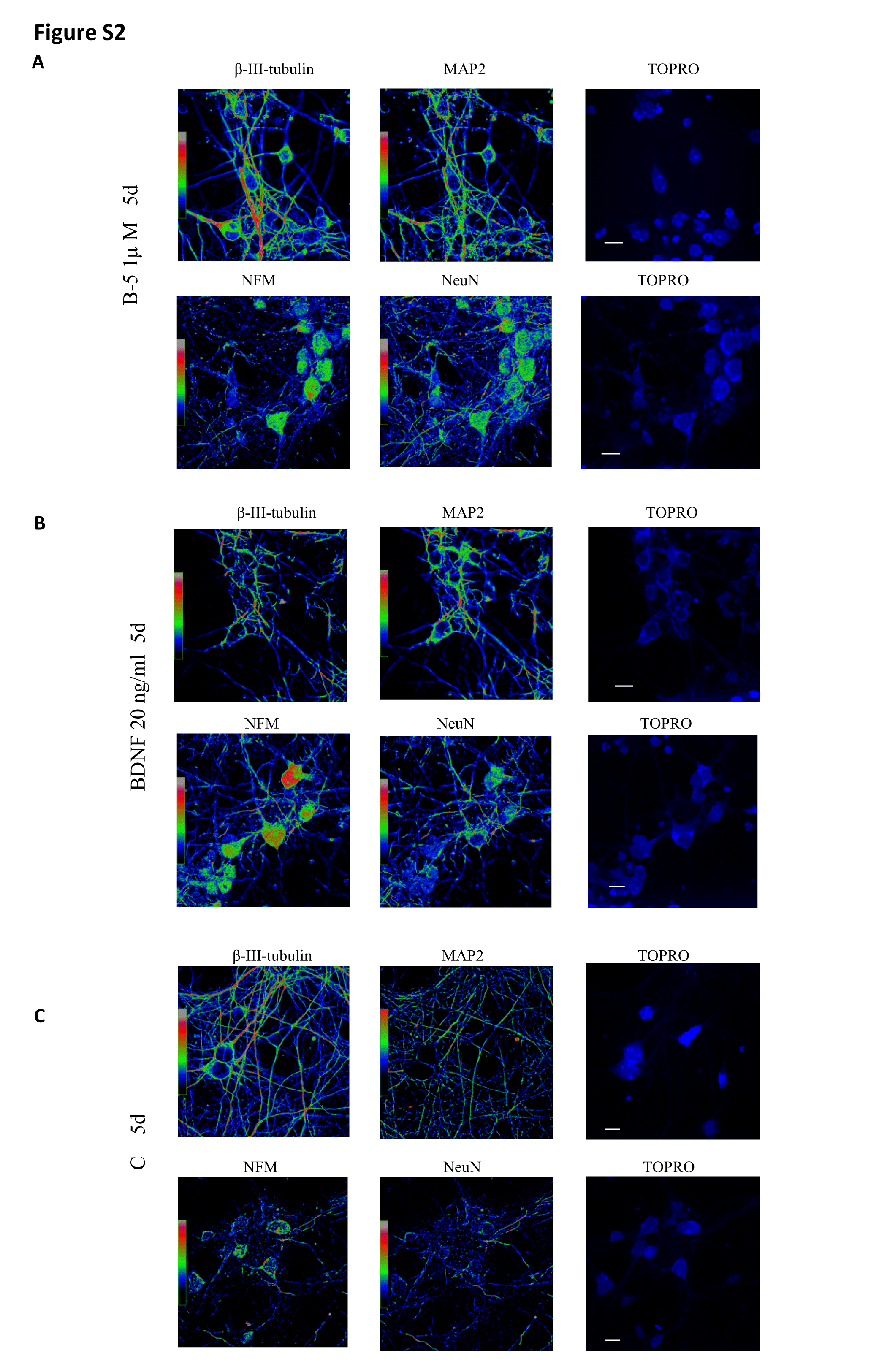

Supplement: Figure S2 — Rainbow images with scale, corresponding to the immunocytochemistry images shown in Figure 3 . The warm colors (like red and yellow) represent the higher level of expression of the neuronal marker analyzed, and the cold colors (like blue) represent the lowest level of expression of the marker. White represents the highest level of expression whereas black represents no expression at all. (A–C) Peptide B-5 and BDNF induce the increase in the level of expression of the neuronal markers β-III-tubulin, MAP2, NFM, and NeuN in E18 primary hippocampal cell cultures as compared to the non-treated control. Representative confocal images in the format of rainbow scale, illustrating double immunolabeling of β-III-tubulin and MAP2 or NFM and NeuN in cells treated for 5 days with Peptide B-5 at 1 µM (A), with BDNF at 20 ng/ml (0.79 nM) (B), and vehicle only (control) (C). TOPRO-3 (blue) was used to stain the nuclei. Magnification bar = 10 µm. To the left side of each image in rainbow format, rainbow color scale is shown. (TIF) [file pone.0053596.s002.tif]

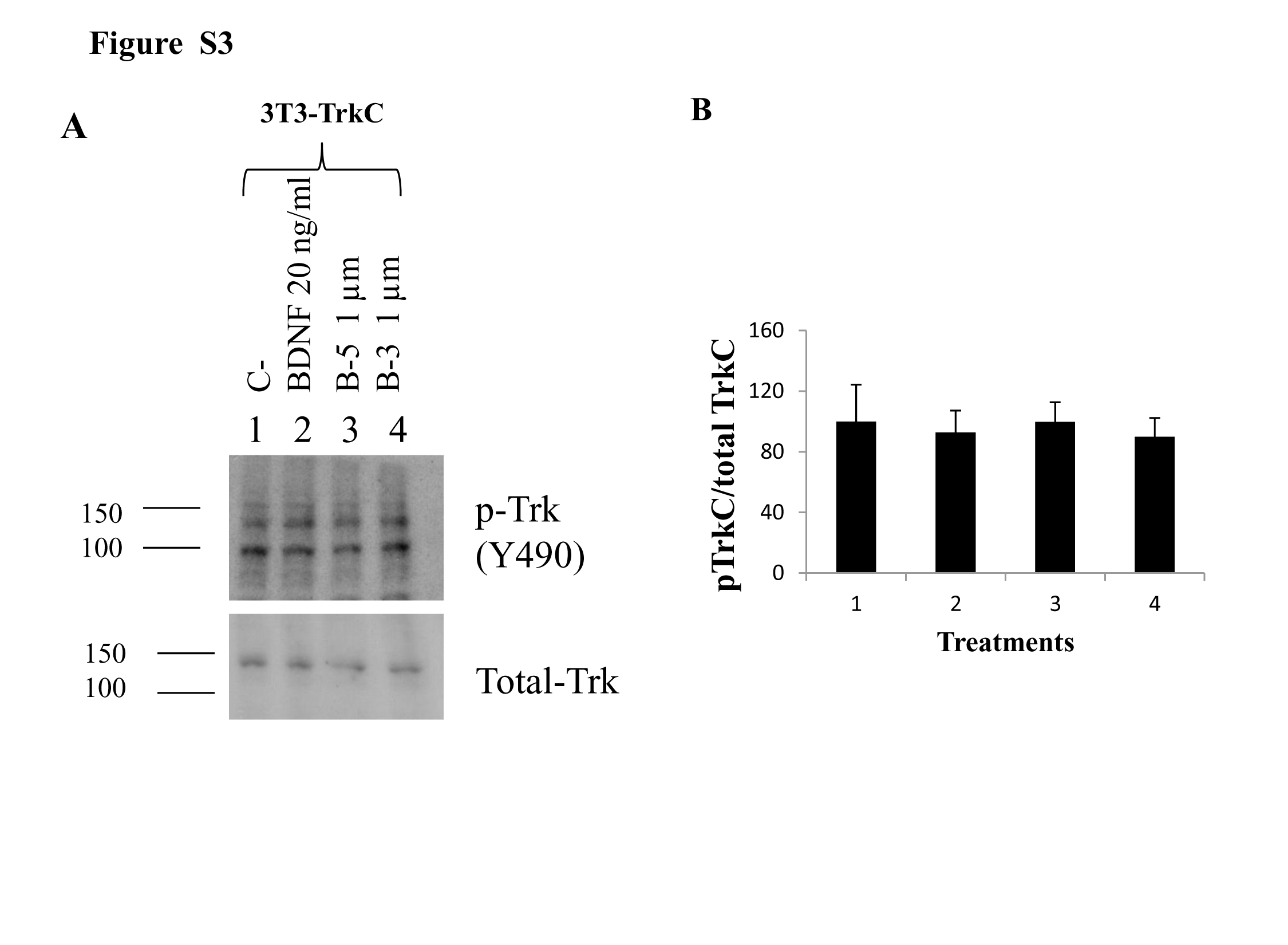

Supplement: Figure S3 — Neither BDNF (20 ng/mL) nor peptides B-5 (1 µM) and B-3 (1 µM) were able to modify the expression of TrkC in TrkC stably-expressing NIH-3T3 fibroblast cells. (A) Western blots of anti-pan-phospho-Trk (Y490) and of total TrkC. GAPDH was used as a loading control. (B) Densitometric quantitation of the Western blots for pTrk normalized to total TrkC (after normalizing TrkC to GAPDH). Data are shown as mean ± standard deviation, n = 3. (TIF) [file pone.0053596.s003.tif]
